# Immunohistochemical Expression of PTEN in Canine Gliomas

**DOI:** 10.3390/ani14142115

**Published:** 2024-07-20

**Authors:** Jéssica Molín, Roberto José-López, Gustavo A. Ramírez, Martí Pumarola

**Affiliations:** 1 Departament Ciència Animal, Campus Agroalimentari, Forestal i Veterinari, Universitat de Lleida, 25198 Lleida, Spain; gustavo.ramirez@udl.cat; 2 Division of Small Animal Clinical Sciences, School of Biodiversity, One Health and Veterinary Medicine, College of Medical, Veterinary and Life Sciences, University of Glasgow, Glasgow G61 1QH, UK; roberto.joselopez.bcn@gmail.com; 3 Neurology and Neurosurgery Service, Southfields Veterinary Specialists, Part of Linnaeus Veterinary Ltd., Basildon SS14 3AP, UK; 4 Unitat de Patologia Murina i Comparada, Departament de Medicina i Cirurgia Animals, Facultat de Veterinària, Universitat Autònoma de Barcelona, 08193 Barcelona, Spain; marti.pumarola@uab.cat

**Keywords:** astrocytoma, dog, high grade, immunohistochemistry, oligodendroglioma, PTEN, PI3K/Akt/mTor, signaling pathways, undefined glioma

## Abstract

**Simple Summary:**

PTEN is a critical tumor suppressor gene that plays a vital role in regulating cell proliferation, migration, and survival. The loss of PTEN function, either by genetic alterations or decreased protein expression, is frequent in human gliomas and has been correlated with tumor progression, grade, therapeutic resistance, and decreased overall survival in patients with glioma. This study investigates the immunohistochemical expression of PTEN in canine gliomas to evaluate possible alterations, as those reported in human gliomas. Our research demonstrates for the first time a variable reduction in PTEN protein expression in high-grade canine gliomas, particularly in astrocytomas. These observations are in line with those reported in human gliomas and provide a rationale for future studies regarding abnormalities in PTEN expression and PI3K/Akt/mTor pathway in canine gliomas, to evaluate its prognostic and therapeutic implications.

**Abstract:**

Phosphatase and tensin homolog (PTEN) is a critical tumor suppressor gene with a vital role in regulating cell proliferation, migration, and survival. The loss of PTEN function, either by genetic alterations or decreased protein expression, is frequent in human gliomas and has been correlated with tumor progression, grade, therapeutic resistance, and decreased overall survival in patients with glioma. While different genetic mutations in PTEN gene have been occasionally reported in canine gliomas, no alterations in protein expression have been reported. This study investigates the immunohistochemical expression of PTEN in canine gliomas to evaluate possible alterations, as those reported in human gliomas. Immunohistochemical PTEN expression and pattern distribution were analyzed in 37 spontaneous canine gliomas. Among gliomas, 52.6% cases showed high PTEN expression and 48.6% displayed reduced (13.5%) or highly reduced (35.1%) immunopositivity. Most oligodendrogliomas showed high expression (73.7%), while the majority of astrocytomas (69.2%) showed a reduced or highly reduced expression. A reduced PTEN expression was mostly associated with a heterogeneous loss of PTEN immunopositivity. These observations are in line with those reported in human gliomas and provide a rationale for future studies regarding abnormalities in PTEN expression and PI3K/Akt/mTor pathway in canine gliomas, to evaluate its prognostic and therapeutic implications.

## 1. Introduction

Glioma is the second most commonly diagnosed primary central nervous system (CNS) neoplasm of dogs, accounting for over 35% of all canine intracranial neoplasms [1,2,3]. Epidemiologic data on glioma in dogs indicate a median age at diagnosis of 8 years and a male predilection (incidence ratio of 1.28–1.53 for males/females) [1,4]. Over 50% of all canine gliomas occur in certain brachycephalic breeds, with the Boston Terrier, Bulldog, and Boxer being the most represented. Intracranial gliomas are far more frequent than spinal gliomas, with most of the lesions located within the fronto-olfactory, temporal, and parietal regions of the cerebral hemispheres [1,2,3]. Historically, the diagnosis of canine gliomas has been based on World Health Organization (WHO) guidelines. However, this outdated classification has been replaced by a more comprehensive grading system, which divides canine gliomas into three broad categories of oligodendroglioma, astrocytoma, and undefined glioma, each of which are further classified into low-grade and high-grade tumors [4,5]. Among them, high-grade tumors are more frequent than low-grade ones, and oligodendroglioma is the most commonly reported (~70% of all canine gliomas), whereas astrocytoma and undefined glioma are less represented [1,4,5].

Canine gliomas show many epidemiological, clinical, and molecular similarities with human glioma, demonstrating their suitability for comparative oncology studies to advance the understanding of glioma pathogenesis, clinical behavior, response to treatment, and the identification of new therapeutic targets [1,4,6,7,8,9]. Advantages of spontaneous canine glioma as a robust intermediate model for human glioma include anatomical characteristics of canine brain, with a relevant blood–brain barrier, natural coevolution of tumor and surrounding microenvironment, intratumoral heterogeneity, and an intact immune system [1,3,4,6,7,8,9,10]. Although our understanding of the molecular landscape of canine gliomas has greatly expanded in the last few years, with several studies demonstrating similarities and differences to human glioma, further investigations regarding abnormalities in major molecular pathways such as TP53, cell cycle regulators, and core members of major signaling pathways involved in both human and canine gliomagenesis and progression are required [9,10,11].

Phosphatase and tensin homolog (PTEN) is a dual lipid and protein phosphatase that acts as a major negative regulator of the phosphoinositide 3 kinase (PI3K)/AKT/mechanistic target of rapamycin (mTOR) pathway, with a key role in controlling a wide range of essential cellular processes including cell proliferation, growth, survival, and metabolism [12,13,14,15,16]. PTEN blocks PI3K by dephosphorylating phosphatidylinositol (PI)-3,4,5-triphosphate (PIP3) to PI-4,5-bisphosphate (PIP2), thus counteracting PI3K function and subsequent AKT/mTor activation [12,13,14,15,16]. The loss of PTEN function by genetic alterations and/or epigenetic, transcriptional, and post-translational mechanisms is an important event involved in tumor malignancy and progression in many different human cancers including glioma, melanoma, and breast, prostate, lung, colon, or endometrial carcinoma, among others [12,13,14,15,16]. The loss of PTEN function in human gliomas is very frequent, particularly in glioblastoma (GB), with PTEN protein loss reported in between 65 and 69% of cases and has been associated with tumor grade, immunosuppressive tumor microenvironment, decreased overall survival time, and resistance to chemotherapy or radiotherapy [14,15,16,17,18].

Molecular investigations in canine gliomas have revealed genetic alterations in RTK/RAS/PI3K, RB, p53, and other genes encoding for cell cycle regulators and growth factors, suggesting canine and human glioma share molecular abnormalities involving same major cell signaling pathways [7,9,10,19]. Although several studies have demonstrated the overexpression of a variety of growth factors and their receptors associated with PI3K and the rat sarcoma viral oncogene (RAS) pathways [20,21,22], data related to protein alterations occurring downstream of the receptors are scarce [10,23]. A couple of studies have demonstrated an increased expression of phospho-ERK ½, mitogen-activated protein kinase (MAPK), phospho-Akt, and phospho-mTor in a small group of canine astrocytomas [10,23].

In canine tumors, alterations in PTEN protein expression have been demonstrated in melanoma [24], hemangiosarcoma [25], osteosarcoma [26,27], prostatic carcinomas [28], and mammary tumors [29,30,31,32]. PTEN expression in canine gliomas has been very little investigated. Genetic mutational studies in 83 canine gliomas (46 oligodendrogliomas, 31 astrocytomas, and 6 undefined gliomas) demonstrated PTEN homozygous deletion and arm-level aneuploidy in PTEN regions in 3% and 14% of cases, respectively [33], while additional investigations reported copy number alterations in PTEN in 15% of canine gliomas [19]. There is only one study addressing PTEN protein expression in canine gliomas by Western blot analysis, showing some variability in PTEN protein expression that may suggest a partial loss of PTEN function as reported for human tumors [10]; however, as stated by the authors, this requires further investigations.

The objective of our study is to investigate the immunohistochemical expression of PTEN in canine gliomas to evaluate whether the loss of PTEN protein is present in different subtypes of high-grade tumors, as well as to further advance the molecular characterization of glioma in dogs.

## 2. Materials and Methods

### 2.1. Case Selection 

Histopathologically confirmed canine gliomas diagnosed between 2010 and 2019 at the Unitat de Patologia Murina i Comparada (UPMiC), Universitat Autonoma de Barcelona (UAB) (*n* = 25), and the School of Biodiversity, One Health and Veterinary Medicine, College of Medical, Veterinary and Life Sciences, University of Glasgow (*n* = 12), were included in the study. All samples were obtained at necropsy within 24 h from death. Written consent for necropsy and histopathological analysis was obtained from all animal owners.

### 2.2. Histology and Morphological Diagnosis

Samples were fixed in 10% neutral buffered formalin. Fixation times varied because of the multicentric and retrospective nature of the study; however, this was always <5 days. Following fixation, the transverse sections of the brain were made, and samples including the tumor area were routinely processed. Morphological evaluation was performed on 4 µm paraffin-embedded sections stained with hematoxylin and eosin. The histological diagnosis of glioma type and grade was performed according to the Comparative Brain Tumor Consortium (CBTC) diagnostic schema [4] by a board-certified veterinary pathologist with expertise in canine neuropathology (M.P.). When available, samples were further evaluated by immunohistochemistry (IHC) for glial fibrillary acidic protein (GFAP) and Olig2 protein.

### 2.3. Immunohistochemistry

The immunohistochemical study for GFAP and Olig2 was performed according to a previously described protocol [34]. PTEN IHC was performed at the Scientific and Technical Service of Immunohistochemistry (Institute for Biomedical Research, Lleida). Tissue blocks were sectioned at thickness of 3 μm, dried for 1 h at 65 °C before pre-treatment procedure of deparaffinization, rehydration and epitope retrieval in the Pre-Treatment Module, PT-LINK (Agilent Technologies-DAKO, Santa Clara, CA, USA), at 95 °C for 20 min in 50 × Tris/EDTA buffer, pH 9. Before staining the sections, endogenous peroxidase was blocked. Sections were incubated with anti-PTEN primary antibody (1:100 dilution, clone 6H2.1; Agilent Technologies-DAKO, Santa Clara, USA). Alignment between canine (NCBI database, accession no. NP_001003192) and mouse PTEN (NCBI database, accession no. NP_032986.1) and rat PTEN (NCBI database, accession no. NP_113794.1) proteins showed 100% homology [30]. Ninety-nine percent homology was found with the human PTEN (NCBI database, accession no. EAW50175.1) [30]. After incubation, the reaction was visualized with the EnVisionTM FLEX + Mouse Linker Detection Kit (Agilent Technologies-DAKO, Santa Clara, USA) for PTEN, using diaminobenzidine chromogen as a substrate. Slides were counterstained with hematoxylin. Sections of canine kidney were included as an external positive control [24,25,30]. Neurons and endothelial cells in each section were used as internal positive controls as described [35,36,37]. Negative controls omitting incubation with the primary antibody were included. 

The immunohistochemical expression of PTEN in one entire tumor section per case was semiquantitative evaluated by two board-certified pathologists (M.P. and J.M.), based on the percentage of positive neoplastic cells and the intensity of the staining in accordance with previously described method for the evaluation of PTEN IHC in human gliomas [36,37]. The percentage of positive cells, evaluated at 100×–200×, was scored as <25%, 25–50%, and >50% [36]. The intensity of the staining, evaluated at 200–400×, was graded as low (negative or very weak staining when compared to endothelial cells, used as internal controls) or high (moderate or intense uniform cytoplasmic staining like that in endothelial cells) [37]. Based on the % of positive cells and the intensity of the staining, cases were classified into three categories: high expression (>50% positive cells with high intensity), reduced expression (>50% positive cells with low intensity or 25–50% positive cells with high intensity), and highly reduced expression (all cases with <25% positive cells or 25–50% positive cells with low intensity) [36]. 

Further, according to the distribution pattern of PTEN expression in tumor sections, evaluated at low-power field, cases were classified as diffuse positive (positive staining in entire section), heterogeneous (positive and negative areas admixed), and diffuse negative (no staining in entire tumor section) [35,38,39,40].

## 3. Results

### 3.1. Clinicopathological Features

The median age of dogs at diagnosis was 7.8 years (range: 2–13 years). There were 16/37 (43.2%) intact females, 6/37 (16.2%) spayed females, 9/37 (24.3%) intact males, and 6/37 (16.2%) neutered males. Most of the cases (30/37; 81%) belonged to brachycephalic dog breeds including 16/37 (43.2%) French Bulldogs, 11/37 (29.7%) Boxers, 2/37 (5.4%) Staffordshire Bull Terriers, and 1/37 (2.7%) Dogues de Bordeaux. The breeds of the remaining 7 dogs (19%) and individual data for each case are detailed in Table 1.

Most gliomas were located in the cerebral hemispheres (25/37), followed by diencephalon (8/37), while only 4/37 (10.8%) were infratentorial (3 mesencephalon, 1 cerebellum). Within the cerebral hemispheres, 9/25 (36%) tumors were in the fronto-olfactory region, 11/25 (44%) in the temporal cortex, 4/25 (16%) in the parietal cortex, and 1/25 (4%) in the occipital cortex.

Of the 37 gliomas diagnosed following the application of the revised diagnostic scheme for canine gliomas, 19 (51.4%) were classified as high-grade oligodendrogliomas (HO), 13 (35.1%) as high-grade astrocytomas (HA), 4 (10.8%) as high-grade undefined gliomas (HU), and only 1 (2.7%) case corresponded to a low-grade oligodendroglioma (LO). Astrocytomas and oligodendrogliomas were characterized by their typical histological features and, when required, further classified based on immunoreactivity against GFAP and Olig2 on >50% of neoplastic cells, respectively.

### 3.2. PTEN Immunohistochemistry

**Figure 1:**
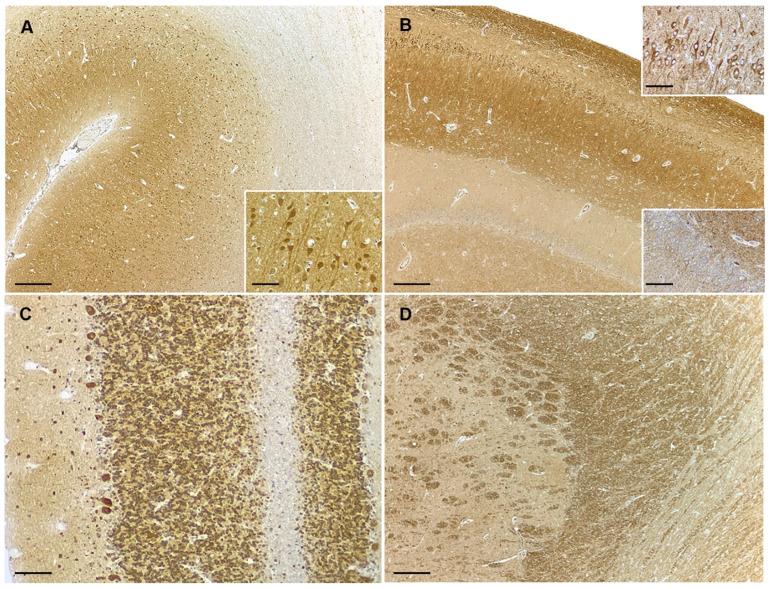
PTEN expression in control canine brain sections. (**A**) Cerebral cortex. High cytoplasmic and nuclear PTEN expression in cortical neurons (inset). Gray matter neuroparenchyma shows diffuse positivity of lower intensity than neuronal bodies but higher than in the white matter of corona radiata. (**B**) Hippocampus. Pyramidal neurons in cornu Ammonis show intense PTEN cytoplasmic stain (upper corner inset), while most neurons within gyrus dentatus are negative or weakly positive (lower corner inset). (**C**) Cerebellum. Neuronal bodies in molecular, Purkinje, and granular cell layer of cerebellar cortex show intense cytoplasmic with or without nuclear PTEN positivity. (**D**) Strong positive PTEN staining is present in some white matter tracts within the capsula interna/striatum. Paraffin sections counterstained with hematoxylin; bars = 250 µm. (**A**,**B**,**D**), 100 µm. (**C**), 50 µm, all insets.

In canine kidney, strong PTEN expression was observed in glomeruli. In non-tumoral tissue from different brain regions contained in stained sections, PTEN immunoreactivity was mostly observed in neurons and vascular endothelial cells, which were used as a positive internal control of the stain. PTEN-positive cells showed moderate-to-strong diffuse cytoplasmatic positivity with or without nuclear staining. Neuronal PTEN positivity was variable among different brain areas, being most prominent in some neuronal populations within cerebral cortex, hippocampus, cerebellum, and brainstem nuclei (Figure 1A–C). Glial cells were multifocally positive. The neuropil in the gray matter showed a diffuse background staining, being less intense than that in neuronal bodies (Figure 1A). The white matter showed diffuse immunopositivity of variable intensity depending on the examined area, being weaker than in cerebral and cerebellar cortex while particularly prominent within the capsula interna (Figure 1A,C,D).

**Figure 2:**
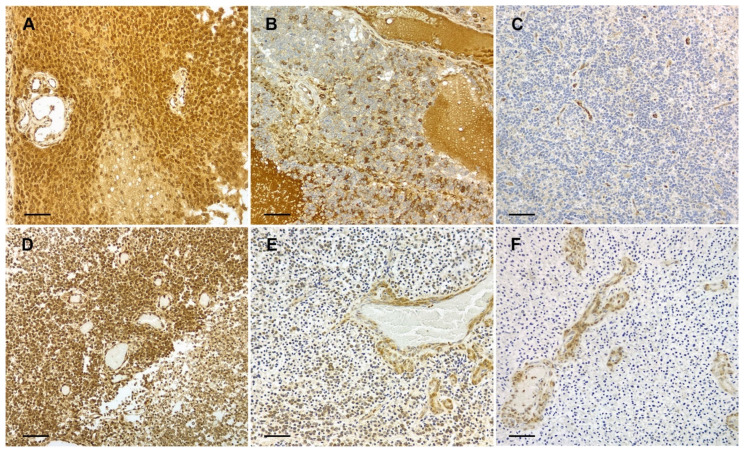
PTEN expression in canine gliomas. (**A**–**C**) High-grade astrocytoma with high and homogeneous positive (**A**), reduced and heterogeneous (**B**), and highly reduced and diffuse negative (**C**) PTEN expression. (**D**–**F**) High-grade oligodendroglioma with high (**D**), reduced (**E**), and highly reduced (**F**) PTEN expression. (**A**–**F**) Intense cytoplasmic and nuclear PTEN positivity is present in the endothelial cells of intratumoral blood vessels, which served as an internal positive control. Paraffin sections counterstained with hematoxylin; bars = 50 µm.

Among gliomas, 19/37 (52.6%) cases showed high PTEN expression and 18/37 (48.6%) displayed reduced (5/37; 13.5%) or highly reduced (13/37; 35.1%) immunopositivity (Figure 2A–C). Regarding differences in PTEN expression among the glioma subtypes included in the study, most oligodendrogliomas showed high expression (14/19; 73.7%), while the majority of astrocytomas (9/13; 69.2%) showed a reduced or highly reduced PTEN protein expression (Table 2). PTEN-positive neoplastic cells showed diffuse cytoplasmic staining variably accompanied by nuclear positivity (Figure 2). Immunohistochemical PTEN staining results for each individual case are provided in Supplementary Table S1.

**Figure 3:**
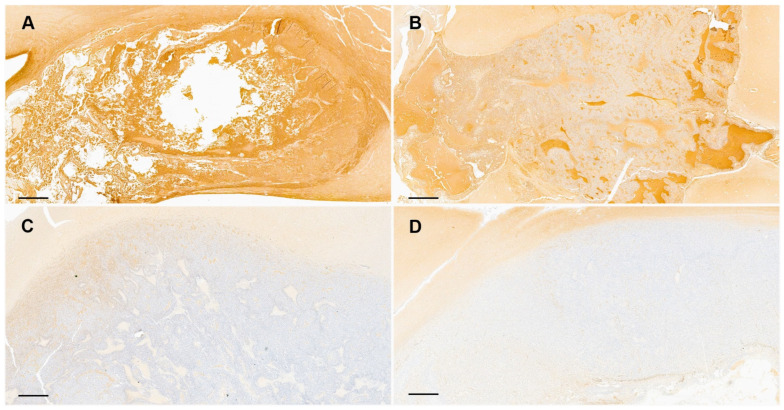
Topographic intratumoral PTEN immunohistochemical expression in canine gliomas. (**A**) Diffuse homogeneous positive high-grade oligodendroglioma with high PTEN expression. (**B**,**C**) Heterogeneous positivity in high-grade astrocytomas with reduced (**B**) and highly reduced (**C**) PTEN expression. (**D**) Diffuse PTEN negative high-grade astrocytoma. Hematoxylin-counterstained sections; bars = 1000 µm.

Regarding the intratumoral topographic distribution of PTEN immunostaining in tumor sections, 15/37 (40.5%) cases showed a diffuse positivity, corresponding all of them to gliomas with high PTEN expression (19/37) (Figure 3A). A heterogeneous pattern was present in 16/37 (46%) cases, 4 with high expression and 12 with reduced or highly reduced PTEN immunostaining (Figure 3B,C). In some of the cases with heterogeneous pattern PTEN-positive areas were mostly confined to the periphery of the tumor, while in others, positive and negative areas were multifocally admixed in a mosaic pattern. The diffuse negative pattern was less frequent, being only observed in 5/18 cases with highly reduced PTEN expression, representing 5/37 (13.5%) of all included gliomas (Figure 3D).

**Figure 4:**
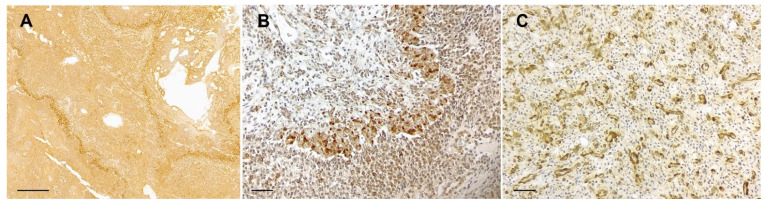
PTEN-immunostained canine gliomas. (**A**–**C**) Representative photomicrographs of the prominent PTEN immunopositivity present in areas of microvascular proliferation in high-grade oligodendrogliomas with high PTEN expression (**A**,**B**) and in an undefined glioma with reduced and heterogeneous PTEN expression (**C**). Paraffin sections counterstained with hematoxylin; bars = 500 µm. (**A**), 50 µm. (**B**,**C**).

Additional observations on intratumoral PTEN immunostaining included prominent endothelial PTEN positivity in areas of microvascular proliferation (Figure 4).

## 4. Discussion

Alterations in PTEN gene/protein levels in human gliomas have been demonstrated as one of the most important mechanisms involved in the constitutive activation of PI3K/Akt/mTor signaling pathway leading to tumor progression, decreased overall survival, and therapeutic resistance in patients with glioma [12,14,15,16,17]. Although molecular alterations in the PI3K/Akt/mTOR pathway have been increasingly recognized in canine gliomas, reflecting similarities to the human counterpart, studies addressing abnormalities in PTEN expression are scarce. While different genetic mutations in the PTEN gene have been reported in 3–15% of canine gliomas [19,33], the Western blot analysis of protein expression failed to show clear alterations [10]. This is the first study assessing PTEN protein expression by IHC in canine gliomas and demonstrating its loss in a subset of high-grade gliomas, particularly in astrocytomas, as reported in the human counterpart.

The epidemiological characteristics of glioma cases included herein are in line with those previously reported [1,2,3,4]. Most of them (81%) occurred in brachycephalic dog breeds belonging to the same phylogenetic clade, supporting glioma pronounced predilection for these breeds [1,2,3,4]. Although a male sex predilection for canine gliomas has been described, females were most represented herein. This discrepancy could be related to the relatively low number of cases included in our study. Glioma type and grade relative frequency in this study are consistent with data from the prior literature, indicating that oligodendroglial and high-grade tumors are the most prevalent in dogs [1,2,3,4].

The immunohistochemical expression of PTEN in canine kidney was equivalent to that reported by other authors [24,25,31], whereas in control brain tissues, it was observed in neurons and endothelial cells, as described in rodents and human brain [41,42,43]. Both normal and tumor cells showed cytoplasmic or cytoplasmic and nuclear PTEN positivity. Although in earlier studies, PTEN was reported to be a protein exclusively localized in the cytoplasm; nowadays, it is clear that PTEN can be both cytoplasmic and nuclear, as widely demonstrated in normal canine tissues and tumors such as hemangiosarcoma, melanoma, and mammary carcinomas [24,27,28,30], as well as in human gliomas [17,41,44]. PTEN has very distinct growth-regulatory roles in the cytoplasm and the nucleus. In the cytoplasm, it has intrinsic lipid phosphatase activity that negatively regulates the cytoplasmic PI3K/Akt pathway, while nuclear PTEN displays important Akt-independent growth-suppressing activities related to chromosome stability, DNA repair, and cell cycle regulation through mechanisms which are still not completely understood [13,15,17]. 

Our results demonstrate a variable reduction in PTEN protein expression in almost 49% of canine gliomas, particularly in astrocytomas with 69.2% of included cases affected. These observations are in line with the reported loss of PTEN immunolabeling in a variable percentage (30%−80%) of human gliomas depending on the type and grade of tumors included in the study [35,36,37,38,39,40,41,44,45,46,47,48]. The loss of PTEN function in human gliomas is more frequent in high-grade than low-grade tumors, and in those from astrocytic origin, especially in GB, reflecting PTEN importance in tumor malignancy and progression [36,39,45,46,48]. Interestingly, it has been seen that, when mTOR signaling suppression occurs, apoptosis is observed in tumor cells, and astrocytoma is transformed into oligodendroglioma. Thus, PTEN loss and subsequent activation of Akt signaling leading to mTOR upregulation appear vital for survival and viability of GB cells as well as for preserving astrocytic features [16].

Three patterns of PTEN immunostaining were observed among glioma cases included in this study, a diffuse positive pattern, an heterogenous pattern, and a diffuse negative pattern. The same three patterns have been described in a similar frequency by previous studies in human gliomas, with the heterogeneous pattern as the most frequently reported in GBs, followed by homogeneous positive and less frequently diffuse negative tumors [35,38,39,40,46,47]. Heterogeneous PTEN immunostaining have also been reported in canine mammary and prostatic carcinomas, hemangiosarcomas, and osteosarcomas [25,26,28,30,32]. Intratumoral heterogeneous PTEN protein immunoexpression in human GBs is a well-known phenomenon, ideally suited as a basis for the evaluation of the molecular abnormalities underlying PTEN expression. While the multifocal loss of PTEN expression has been correlated with some genetic and epigenetic abnormalities including gene methylation, silencing, and loss of heterozygosity by some authors [38,40], it is well known that the mutational inactivation of the PTEN gene in GB is less frequent than the loss of protein expression [14,15,16,17,18]. Previous studies of genetic PTEN abnormalities in canine gliomas revealed different mutations including homozygous deletion, arm-level aneuploidy, and copy number alterations in 3%, 14%, and 15% of cases, respectively [19,33]. Thus, the rate of PTEN mutations reported for canine gliomas is lower than the proportion of cases showing reduction in PTEN immunopositivity in our study (49%), resembling observations in human gliomas. Possible reasons for discrepancies between gene status and protein levels might rely on the diversity of mechanisms that may coexist to attenuate PTEN expression in tumor cells which, besides genetic alterations, include epigenetic mechanisms, post-translational modifications, protein–protein interactions, and increased protein degradation [12,17]. Further studies evaluating the presence of PTEN genetic abnormalities and immunohistochemical PTEN expression are required to evaluate possible correlations between gene alterations and protein loss in canine gliomas.

To the authors’ knowledge, there is only one study investigating PTEN protein expression in canine gliomas by Western blot, which evidenced a variable expression of PTEN protein among different canine gliomas and normal CNS tissues [10]. Although no significant differential PTEN expression between control tissues and tumors were observed, results suggested the loss of protein expression in a subset of canine gliomas and demonstrated an increased expression of phospho-AKT and phospho-mTor in a small group of canine astrocytomas, also corroborating previous observations [10,23]. These observations are consistent with the increased frequency of PTEN protein loss in high-grade gliomas, particularly astrocytomas, encountered by us, which would lead to the increased activation of PI3K/AkT/mTor pathway with consequent activation of its downstream effectors as reported [10,23]. The discrepancies between obtained results of PTEN expression in canine gliomas in our immunohistochemical study and those previously observed by Western blot could mainly relate to the different techniques employed for PTEN evaluation between the studies. Also, we suspect that the intratumoral heterogeneity of PTEN expression in canine gliomas, as demonstrated herein, as well as the variable non-tumoral PTEN protein expression in both CNS normal tissues and intratumoral blood vessels might have contributed to the lack of clear detectable loss of PTEN protein expression by Western blot in the previous study. In this sense, IHC appears to be a better approach for the evaluation of PTEN protein expression in canine tumors than immunoblotting, as it allows the evaluation of the topographic distribution of the protein within the tumor and its expression in tumor cells, excluding protein expression in non-tumoral tissue. Regarding non-tumoral PTEN protein expression, some of the included cases showed a very prominent PTEN staining in endothelial cells in areas of microvascular proliferation, which was of higher intensity than that in tumor cells or in endothelium of non-proliferating capillaries, like that occasionally reported in human gliomas [39]. PTEN plays a critical role in endothelial cells during angiogenesis by regulating cell proliferation and migration, preventing excessive proliferation by ensuring balanced angiogenic signals, modulating vascular endothelial growth factor (VEGF) signaling, and ensuring vessel maturation and stability [49,50]. These functions underscore PTEN’s importance in both initiating and regulating angiogenesis. We hypothesize that the high PTEN expression in areas of microvascular proliferation in some of the studied cases might be related to the antiangiogenic functions of PTEN in promoting neovessels stability and maturation. 

Reduced or loss of PTEN function in human gliomas is involved in tumor progression by increasing cell survival, polarity, and motility, and, in addition to tumor grade, it has been correlated with decreased survival time and resistance to radiotherapy, chemotherapy, and immunotherapy in patients with glioma [14,15,16,17,18]. Recent studies have demonstrated an important role of PTEN expression in controlling tumor immune microenvironment (TIME) and its contribution to immunomodulatory therapy resistance in patients with glioma [51,52,53,54,55]. PTEN exerts its immunomodulatory roles by inducing changes in tumor-associated immune cell infiltrates and by increasing the expression of immunosuppressive molecules in tumor cells, thus influencing both innate and adaptative tumoral immune responses. Several studies confirmed that the loss of PTEN expression in human gliomas induce changes in cytokine secretion pattern, promoting an immune-suppressive microenvironment characterized by reduction in tumor infiltrating lymphocytes (TILs) and increased immune cell populations that can promote tumor progression, such as regulatory T cells (Tregs) and M2 macrophages [51,52,53,54,55]. Moreover, PTEN loss increases PD-L1 expression in glioma cells by accelerating protein synthesis though PI3K-dependent activation and elevated programmed cell death-ligand 1 (PD-L1) protein translation rate [55]. In fact, PTEN mutations have been correlated with a lack of sensitivity to anti-PD-1/PD-L1 therapy and, therefore, combinatorial strategies between PD-1/PD-L1 inhibitors and PI3K/AKT targeting drugs are being proposed to overcome resistance to immune checkpoint inhibition in human gliomas [53,55]. Recent studies on immune cell infiltration in canine gliomas have demonstrated increased numbers of CD3 + and FOXP3 + TILs in high-grade tumors, particularly in astrocytomas, and M2 polarization of macrophagic infiltrates in high-grade gliomas, mirroring similar TIME to that described for human gliomas [34,56]. Additional studies by our group have shown an increased expression levels of PD-L1 in high-grade canine gliomas, being higher in those from an astrocytic origin [57], similar to human gliomas [55,58]. The increased loss of PTEN expression encountered in high-grade astrocytomas in this study could be in line with the more pronounced presence of TIME modulatory elements reported for canine astrocytomas.

## 5. Conclusions

Our results characterize PTEN protein immunohistochemical expression in canine gliomas for the first time and demonstrate a variable reduction in its expression in a subset of high-grade canine gliomas, being more frequent in those from an astrocytic origin. PTEN expression in canine gliomas might be heterogeneous, homogeneously positive, or homogeneously negative, with the first distribution pattern being the most frequent, while diffuse immunonegative tumors are rarely seen. These observations are in line with those reported in human gliomas and provide a rationale for future studies regarding abnormalities in PTEN expression and PI3K/Akt/mTor pathway in canine gliomas. Further investigations to evaluate the possible associations between PTEN loss of function and tumor grade, TIME, and whether PTEN status might influence progression and response to radiotherapy, chemotherapy, or immunotherapy in canine patients are required to assess the prognostic and therapeutic implications of decreased PTEN expression in canine gliomas.

## Figures and Tables

**Table 1:** Clinical features and histopathologic classification of included cases.

Case	Breed	Sex	Age (Years)	Tumor Location	Diagnosis
1	Boxer	M	8.0	Diencephalic	HA
2	Boxer	FN	4.8	Infratentorial (mesencephalon)	HA
3	Boxer	M	9.0	Infratentorial (cerebellum)	HA
4	French bulldog	FN	10.0	Hemispheric (parietal)	HA
5	Boxer	F	8.3	Hemispheric (temporal)	HA
6	Staffordshire bull terrier	M	11.7	Hemispheric (fronto-olfactory)	HA
7	Boxer	MN	8.4	Infratentorial (mesencephalon)	HA
8	Boxer	M	9.8	Hemispheric (fronto-olfactory)	HA
9	Polish Tatra Sheepdog	FN	7.0	Hemispheric (parietal)	HA
10	French bulldog	MN	8.0	Hemispheric (temporal)	HA
11	German Shepherd	M	5.0	Hemispheric (occipital)	HA
12	French bulldog	F	7.0	Infratentorial (mesencephalon)	HA
13	Boxer	FN	8.0	Hemispheric (temporal)	HA
14	French bulldog	F	2.0	Diencephalic	HO
15	Yorkshire terrier	F	13.0	Hemispheric (fronto-olfactory)	HO
16	French bulldog	F	8.2	Diencephalic	HO
17	Dogue de Bordeaux	M	3.7	Hemispheric (parietal)	HO
18	Border Terrier	MN	5.0	Diencephalic	HO
19	West Highland white terrier	MN	9.3	Hemispheric (parietal)	HO
20	Staffordshire bull terrier	MN	7.5	Diencephalic	HO
21	Boxer	FN	8.6	Hemispheric (fronto-olfactory)	HO
22	German Shepherd	FN	7.5	Hemispheric (temporal)	HO
23	French bulldog	F	3.2	Hemispheric (fronto-olfactory)	HO
24	Boxer	F	6.3	Hemispheric (fronto-olfactory)	HO
25	French bulldog	F	9.0	Hemispheric (temporal)	HO
26	French bulldog	F	7.5	Hemispheric (fronto-olfactory)	HO
27	Boxer	F	7.7	Hemispheric (temporal)	HO
28	French bulldog	M	10.0	Hemispheric (temporo-parietal)	HO
29	French bulldog.	F	6.0	Hemispheric (temporal)	HO
30	French bulldog	MN	9.0	Hemispheric (temporal)	HO
31	French bulldog	F	9.0	Hemispheric (fronto-olfactory)	HO
32	Boxer	F	7.5	Diencephalic	LO
33	French bulldog	F	10.2	Hemispheric (temporal)	HO
34	French bulldog	M	8.0	Hemispheric (temporal)	HU
35	French bulldog	F	9.0	Hemispheric (frontal)	HU
36	Mongrel	F	6.5	Diencephalic	HU
37	French bulldog	M	10.4	Diencephalic	HU

F: female; FN: female neutered; M: male; MN: male neutered; HO: high-grade oligodendroglioma; HA: high-grade astrocytoma; HU: high-grade undefined glioma; and LO: low-grade oligodendroglioma.

**Table 2:** Immunohistochemical expression of PTEN in canine gliomas.

PTEN IHC	HO	HA	HU	LO	TOTAL
Expression					
High	12/19 (63.2%)	4/13 (30.8%)	2/4 (50%))	1/1 (100%)	19/37 (52.6%)
Reduced	3/19 (15.8%)	2/13 (15.4%)	0/4 (0%)	0/1 (0%)	5/37 (13.5%)
Highly reduced	4/19 (21%)	7/13 (53.8%)	2/4 (50%)	0/1 (0%)	13/37 (35.1%)
Pattern					
Diffuse positive	10/19 (52.6%)	4/13 (30.8%)	0/4 (0%)	1/1 (100%)	15/37 (40.5%)
Heterogeneous	8/19 (42.1%)	6/13 (46.1%)	3/4 (75%)	0/1 (0%)	17/37 (46%)
Diffuse negative	1/19 (5.3%)	3/13 (23.1%)	1/4 (25%)	0/1 (0%)	5/37 (13.5%)

HO: high-grade oligodendroglioma; HA: high-grade astrocytoma; HU: high-grade undefined; LO: low-grade oligodendroglioma.

## Data Availability

The data presented in this study are available on request from the corresponding author.

## Supplementary Materials

The following supporting information can be downloaded at: https://www.mdpi.com/article/10.3390/ani14142115/s1, Table S1: Immunohistochemical results of evaluated canine gliomas.
