# Supplementary material for: Immunohistochemical Expression of PTEN in Canine Gliomas
Source: Animals (Basel). 2024 Jul 20;14(14):2115. doi: 10.3390/ani14142115 (PMC11273977; doi:10.3390/ani14142115)
Supplement: Supplementary file 1 [file animals-14-02115-s001.zip › animals-3095700-supplementary.pdf]

Table S1. Individual results of PTEN immunohistochemistry in evaluated canine gliomas

Case	Diagnosis	% cells	Intensity	Result (%, intensity)	Intratumoral pattern	Cellular pattern
1	HA	<25	High	Highly reduced	Heterogeneous	C > CN
2	HA	25-50	High	Reduced	Heterogeneous	C > CN
3	HA	<25	High	Highly reduced	Heterogeneous	C > CN
4	HA	<25	High	Highly reduced	Heterogeneous	C >CN
5	HA	>50	Low	Reduced	Heterogeneous	CN >C
6	HA	<25	High	Highly reduced	Heterogeneous	C > CN
7	HA	>50	High	High expression	Diffuse positive	C > CN
8	HA	<25	High	Highly reduced	Diffuse negative	C
9	HA	<25	High	Highly reduced	Diffuse negative	CN >C
10	HA	<25	High	Highly reduced	Diffuse negative	C > CN
11	HA	>50	High	High expression	Diffuse positive	C > CN
12	HA	>50	High	High expression	Diffuse positive	C > CN
13	HA	>50	High	High expression	Diffuse positive	C > CN
14	HO	>50	High	High expression	Diffuse positive	CN > C
15	HO	25-50	High	Reduced	Heterogeneous	C > CN
16	HO	>50	High	High expression	Diffuse positive	C > CN
17	HO	25-50	High	Reduced	Heterogeneous	CN > C
18	HO	25-50	High	Reduced	Heterogeneous	C > CN
19	HO	<25	High	Highly reduced	Heterogeneous	C > CN
20	HO	>50	High	High expression	Heterogeneous	C > CN
21	HO	>50	High	High expression	Diffuse positive	C > CN
22	HO	>50	High	High expression	Diffuse positive	C > CN
23	HO	>50	High	High expression	Diffuse positive	C
24	HO	<25	High	Highly reduced	Heterogeneous	C > CN
25	HO	>50	High	High expression	Diffuse positive	C > CN
26	HO	>50	High	High expression	Diffuse positive	C > CN
27	HO	>50	High	High expression	Diffuse positive	C > CN
28	HO	>50	High	High expression	Heterogeneous	C > CN
29	HO	>50	High	High expression	Diffuse positive	C > CN
30	HO	>50	High	High expression	Heterogeneous	C
31	HO	>50	High	High expression	Diffuse positive	C > CN
32	LO	>50	High	High expression	Diffuse positive	C > CN
33	HO	<25	Low	Highly reduced	Diffuse negative	C
34	HU	>50	High	High expression	Heterogeneous	CN > C
35	HU	<25	High	Highly reduced	Diffuse negative	C
36	HU	<25	Low	Highly reduced	Heterogeneous	C
37	HU	>50	High	High expression	Heterogeneous	C > CN

HA: high-grade astrocytoma; HO: high-grade oligodendroglioma; LO: low-grade oligodendroglioma; HU: high-grade undefined glioma; C: cytoplasmic; CN: cytoplasmic and nuclear
